# Impact of the 2016 Policy Change on the Delivery of MedsCheck Services in Ontario: An Interrupted Time-Series Analysis

**DOI:** 10.3390/pharmacy7030115

**Published:** 2019-08-12

**Authors:** Ahmad Shakeri, Lisa Dolovich, Lori MacCallum, John-Michael Gamble, Limei Zhou, Suzanne M. Cadarette

**Affiliations:** 1 Leslie Dan Faculty of Pharmacy, University of Toronto, Toronto, ON M5S 3M2, Canada; 2 School of Pharmacy, University of Waterloo, Kitchener, ON N2L 3G1, Canada; 3 Department of Family Medicine, McMaster University, Hamilton, ON L8P 1H6, Canada; 4 Banting & Best Diabetes Centre, Faculty of Medicine, University of Toronto, Toronto, ON M5G 2C4, Canada; 5 ICES, Toronto, ON M5T 3M6, Canada; 6 Dalla Lana School of Public Health, University of Toronto, Toronto, ON M5T 3M7, Canada; 7 Eshelman School of Pharmacy, University of North Carolina, Chapel Hill, NC 27599-7355, USA

**Keywords:** community pharmacy services, health policy, interrupted time series analysis, medication reconciliation

## Abstract

MedsCheck (MC) is an annual medication review service delivered by community pharmacists and funded by the government of Ontario since 2007 for residents taking three or more medications for chronic conditions. In 2010, MC was expanded to include patients with diabetes (MCD), home-bound patients (MCH), and residents of long-term care homes (MCLTC). The Ontario government introduced an abrupt policy change effective 1 October 2016 that added several components to all MC services, especially those completed in the community. We used an interrupted time series design to examine the impact of the policy change (24 months pre- and post-intervention) on the monthly number of MedsCheck services delivered. Immediate declines in all services were identified, especially in the community (47%–64% drop MC, 71%–83% drop MCD, 55% drop MCH, and 9%–14% drop MCLTC). Gradual increases were seen over 24 months post-policy change, yet remained 21%–76% lower than predicted for MedsCheck services delivered in the community, especially for MCD. In contrast, MCLTC services were similar or exceeded predicted values by September 2018 (from 5.1% decrease to 3.5% increase). A more effective implementation of health policy changes is needed to ensure the feasibility and sustainability of professional community pharmacy services.

## 1. Introduction

Chronic medications are often critical to an individual’s ability to maintain and improve their health [1]. However, drug therapy problems, such as unnecessary therapy, ineffective dosage, and adverse drug reactions are common [2,3]. Effective strategies to identify and decrease drug therapy problems are important to reduce the potential harms from medication [4]. Community pharmacists are widely accessible and commonly maintain a comprehensive record of an individual’s prescription medications [5]. Community pharmacists are thus well positioned to effectively identify drug therapy problems and prevent adverse drug events [6,7]. Community-pharmacist medication review programs are well-established and publicly funded in Australia, Denmark and the United Kingdom [8,9,10]. Canada has universal Medicare that covers all medically necessary physician services, and at minimum, partial coverage of medications listed on provincial formularies for seniors and specialized vulnerable groups [11]. In April 2007, the government of Ontario launched the MedsCheck program as the first publicly funded community-pharmacy delivered professional service outside of drug dispensation. When it was originally introduced, the MedsCheck (MC) service was a one-on-one annual consultation service in a community pharmacy that provided education and assessed adherence to therapy. The program targeted Ontario residents taking three or more medications for chronic conditions, and pharmacy reimbursement was limited to once every 12 months [12]. The opportunity for pharmacies to be reimbursed for unlimited follow-up services was added in November 2007. In September 2010, the MedsCheck program was expanded to include people with diabetes (MCD), people unable to physically attend a medication review service in a pharmacy due to physical or mental incapability (MedsCheck at Home [MCH]), and residents in long-term care (MCLTC) [13,14]. In addition, unlimited follow-up services within the year were included for MCD and quarterly services were included for MCLTC. MC and MCD annuals and their respective follow-ups are completed in community pharmacies, whereas MCH and MCLTC occur in a person’s place of residence. Pharmacies are reimbursed for these services by submitting a claim to the Ontario Drug Benefit program. Of interest is the fact that all long-term care (LTC) homes are required by legislation to ensure pharmacist participation in quarterly and annual medication reviews with each patient [13].

The Ontario government announced changes to MedsCheck service delivery in July 2016 that were implemented on 1 October 2016. The mandatory changes added several components to each service, including follow-up services, such as requiring written patient consent, structured pharmacist documentation, and the provision of summary information and recommendations to physicians (Table 1) [15]. MCD included an extra set of assessment parameters related to diabetes education and goal-setting, and new requirements that pharmacists have either a certified diabetes educator designation or have gained adequate diabetes management knowledge through a professional continuing education program approved by the Canadian Council on Continuing Education in Pharmacy [13,16]. However, MCD follow-up services were less rigorous, intended for education and did not require a complete medication review. The objective of this study was to estimate the change in the monthly number of MedsCheck services delivered following the 2016 policy change.

We hypothesized that the increased workload accompanying the policy change and added documentation and training requirements for MCD delivery would impact the feasibility of community services, and thus, an immediate decline in the number of services would be identified. However, given that regular medication reviews are required in LTC, we hypothesized that pharmacies would leverage the MCLTC program to meet medication review requirements and thus, changes to the program would have minimal impact on the frequency of MCLTC services.

## 2. Materials and Methods

### 2.1. Study Design

We used an interrupted time-series segmented regression design to study trends in the monthly services delivered [17,18,19]. Interrupted time series is a robust, quasi-experimental approach for evaluating the effects of policy and public health interventions [17,20]. This method models changes in the level (immediate impact) and trend (slope) after a policy change; in our case, we examined the change in monthly MedsCheck services following the 1 October 2016 policy change.

### 2.2. Data Sources

We identified all MedsCheck services from October 2014 to September 2018 based on the product identification number submitted to the Ontario Drug Benefit program (Table 2). Age and sex were identified from the Registered Persons Database. These data were linked using unique encoded identifiers and analyzed at ICES. Records with errors (death date prior to first service date, age <0, missing age or missing sex) were excluded, and duplicate claims in the same month were deleted. The total number of claims over the study period and the proportion of women and mean age of people receiving each service were summarized by MedsCheck type (MC, MCD, MCH or MCLTC) based on their first service date of each type.

### 2.3. Analytical Approach

The primary outcome was the monthly number of MedsCheck claims by MedsCheck service type (MC annual or follow-up, MCD annual or follow-up, MCH, MCLTC annual or quarterly). We used segmented regression to study trends in monthly service delivery, accounting for autocorrelation, non-stationarity and seasonality. Autocorrelation was examined in the data using the Durbin-Watson test [21] and autocorrelation plots, and stationarity was tested using the Dickey–Fuller test [22]. We specified our model to estimate level and trend changes in MedsCheck services in the 24-months pre-implementation and 24-months post-implementation periods, while accounting for seasonality, non-stationarity, and autocorrelation.

*y* = β_0_ + β_1_ × *time* + β_2_ × *implementation* + β_3_ × *time after* + *e* (1)

Equation (1) Generalized equation (autoregressive and moving average terms not included).

Equation (1) includes the generalized equation, where y was the number of MedsCheck services delivered, β_0_ the number of MedsCheck services delivered in October 2014, β1 the monthly trend in MedsCheck service delivery pre-implementation (October 2014–September 2016), β2 the immediate (level) change in monthly MedsCheck services after implementation (October 2016), β3 monthly trend in MedsCheck service delivery post-implementation (October 2016–September 2018), and *e* the random error. The pre-intervention level and linear trends were plotted using model coefficients, and the level and trend predicted values absent of the policy change were calculated for comparisons [23]. Analyses were completed using R 3.5.2 (Vienna, Austria) leveraging the Companion to Applied Regression (car) and Linear and Nonlinear Mixed Effects Models (nlme) packages [24].

## 3. Results

We identified 2,952,434 MC annual or follow-up services, 848,911 MCD annual or follow-up services, 103,591 MCH services, and 1,084,410 MCLTC annual or quarterly services over the 48-month study period, Table 3. The mean age at the first Medscheck service was lowest for MC (mean = 58.3, SD = 17.3) and highest for MCLTC (mean = 82.6, SD = 10.5). Besides MCD, a higher proportion of MedsCheck service recipients were women.

**Figure 1:**
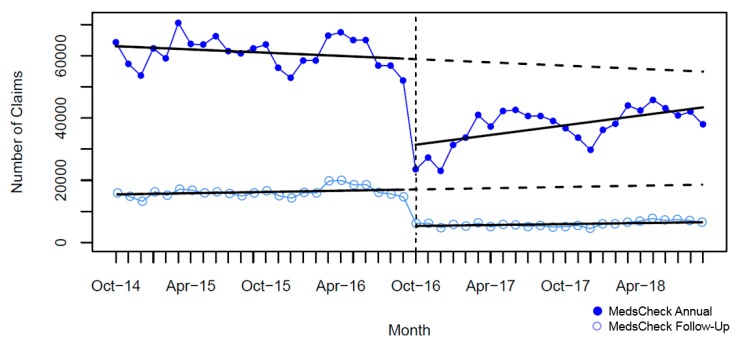
Monthly number of MedsCheck claims relative to the implementation of the October 2016 policy change (vertical dashed line = intervention date). The solid lines show fitted values from the interrupted time series model and the dashed lines show the predicted trend absent the policy change.

**Figure 2:**
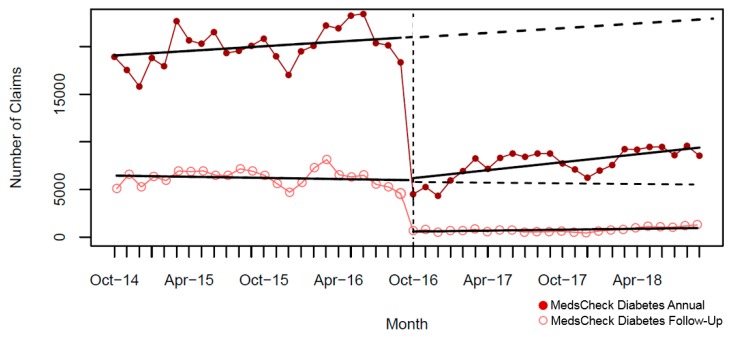
Monthly number of MedsCheck Diabetes claims relative to the implementation of the October 2016 policy change (vertical dashed line = intervention date). Solid lines show fitted values from the interrupted time series model, and dashed lines show the predicted trend absent the policy change.

**Figure 3:**
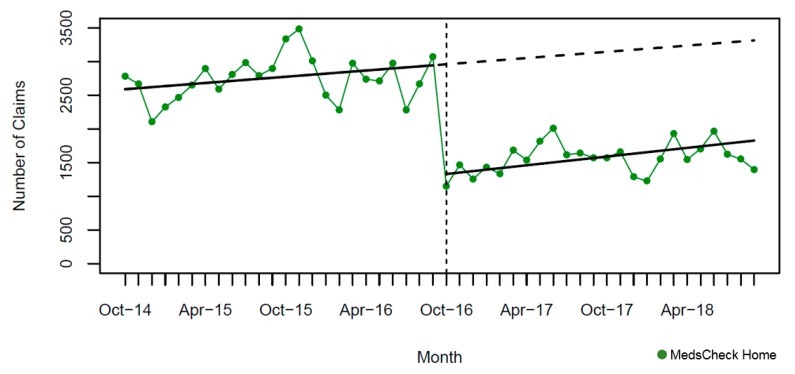
Monthly number of MedsCheck at Home claims relative to the implementation of the October 2016 policy change (vertical dashed line = intervention date). The solid lines show fitted values from the interrupted time series model and the dashed lines show the predicted trend absent the policy change.

**Figure 4:**
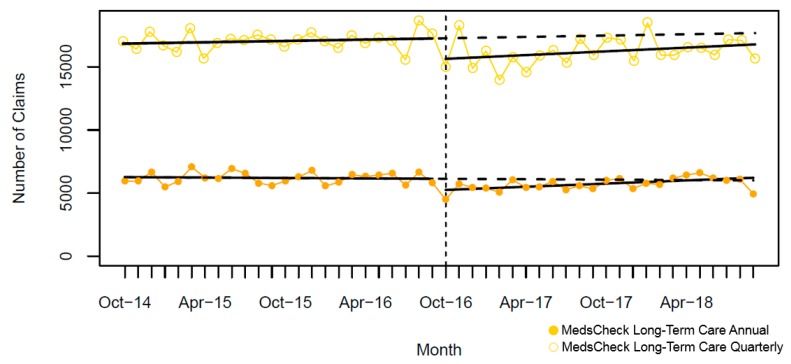
Monthly number of MedsCheck Long Term Care claims relative to the implementation of the October 2016 policy change (vertical dashed line = intervention date). The solid lines show fitted values from the interrupted time series model and the dashed lines show the predicted trend absent the policy change.

In the two years pre-intervention, there was a significant monthly increase for MCD annual and MCH; yet, we observed a slight significant monthly decline for MCLTC annual (Table 4). A significant immediate and large drop in the monthly number of MedsCheck community services was seen after policy implementation (October 2016), ranging from 47% to 64% for MC (Figure 1), 71% to 83% for MCD (Figure 2), and was 55% for MCH (Figure 3). In contrast, a considerable smaller decline was seen for MCLTC, ranging from 9% to 14% (Figure 4). Gradual increases were seen over 24 months post-intervention period, yet remained 21% to 76% lower than predicted for MedsCheck services delivered in the community, particularly for MCD. In contrast, MCLTC services were similar to or exceeded predicted values by September 2018 (from a 5.1% decrease to a 3.5% increase).

## 4. Discussion

MedsCheck was introduced in 2007 as a publicly funded medication review service for patients in the community taking multiple medications, and expanded in 2010 to target other high-risk groups (people with diabetes, home bound and residents of LTC homes) [12,13]. Despite the rapid uptake of these services [14,25], some concerns were raised about quality resulting from the lack of standard documentation or interprofessional coordination [26,27]. In response, the Ontario government consulted the Pharmacy Council—a body that includes representation from the Ontario Pharmacists Association and other stakeholders to provide advice and recommendations related to the pharmacy profession on the development of standardized documentation for the suite of MedsCheck services [28,29,30]. The added service components resulted in an immediate decline in the number of MedsCheck services delivered in the community. The decreases were generally sustained over the subsequent 2 years. However, there was minimal impact on the number of MedsCheck services delivered in LTC. Conceivably, the new requirements may have improved service quality. Future mixed method studies that consider the relative quantity and quality of MedsCheck services pre- and post-policy change are of high interest.

We found that MedsCheck follow-up services remained 61% (MC follow-up) to 76% (MCD follow-up) below predicted 24 months after the policy change. This may partially be attributed to pharmacies being ill-equipped to integrate routine follow-up services into their day-to-day workflow. Reminder systems to identify patients that may benefit from follow-up services are not routine. In addition, the use of part-time staffing and recent pharmacy funding cuts [31,32,33,34] may make it difficult for pharmacists to step away from their dispensing duties and reach out to patients to conduct a follow-up service [35]. Appointment-based pharmacy models are becoming more common in the United States and research demonstrates that appointment-based models improve the number of prescription fills, reduce the number of trips to the pharmacy, improve vaccination rates, and increase the proportion of patients adherent to therapy [36]. The significant impact of the policy change on the number of follow-up services in our study is concerning since monitoring and follow-up with patients on a consistent basis allows for the evaluation of drug therapy effectiveness and adherence, as well as identifying new drug therapy problems [37,38].

The impact on MCH is unexpected given the higher reimbursement ($150 MCH vs. $60 MC annual and $75 MCD annual). However, increased documentation and reporting on top of travel time and expense (i.e., fuel and parking) to visit a patient’s home, medicine cabinet reviews and the removal of unused drugs for proper disposal at the pharmacy [39], may have made the service less feasible. MCH serves an important role in the community as it provides medication review to homebound individuals, typically frail and elderly or living in isolated settings. These patients are often more vulnerable to medication related errors [32]. Managing medications in the home setting is a unique challenge and MCH has the potential to educate patients and their families about medication therapy, and thus ultimately contribute to improved health and quality of life.

Of interest is the fact that the number of monthly MCLTC services changed minimally after the policy change and by 24 months after the policy change, services were above or close to the predicted values. The legislative requirement for annual and quarterly medication reviews together with existing infrastructure logically made MCLTC more feasible, i.e., much of the labour required in the community was already automated in LTC. MCLTC serves an important role since seniors make up about 12% of the Canadian population and 2.6% of all Canadian seniors reside in LTC [40].

Our study had a major strength by leveraging complete population-based level data submitted for reimbursement. However, we were limited in the ability to adjust monthly claims by the number of people eligible to receive MedsCheck services. Drug dispensation for chronic medication is increasing in Ontario [33], therefore, we expect the number of eligible patients for MC and MCH services to be increasing over time [41,42,43]. Similarly, the dispensation of diabetes medications is increasing, indicating an increase in the number of patients eligible to receive MCD services. Finally, the number of LTC beds increased by 2.5%, from 76,982 in 2016 to 78,872 in 2018 [40,44], thus, it is not surprising that we found a 3.5% higher than projected number of MCLTC annual services by September 2018. Despite our inability to adjust claims based on the number of eligible claimants, our results are population-based and compelling given the negligible to slow increase in the number of MedsCheck services over time.

Overall, the 2016 policy change that added several documentation and reporting requirements for MedsCheck services was well-intended to help standardize service delivery yet was followed by a substantive decline in the use of MedsCheck services. Although better standardization in program delivery is desirable, better understanding and pilot testing for feasibility before rolling-out new policies is encouraged. Indeed, leveraging a framework to optimize the design and implementation of new programs has the potential to improve success, value and impact [45,46]. On 25 April 2019, the Ontario Government announced a proposal to “modernize” the MedsCheck program with a sole focus on transitions in care and LTC [47]. The proposed change will lead to the discontinuation of all community MedsCheck services outside of transitions in care. The details have yet to be announced, yet we speculate that the termination of community-based MedsCheck programs relates to the keen interest by the new government to reduce the deficit, coupled with the decline in service delivery and lack of evidence of clinical benefit [48]. For example, the provincial budget notes that “Changing the way pharmacy fees are paid, including a tiered framework for drug mark-up fees; fees paid for filling prescriptions for long-term care home residents, and focusing the MedsCheck program on patients transitioning between health care settings, [will result] in annual savings of over $140 million by 2021–22” [49]. The decision to continue supporting MedsCheck services around transitions in care may relate to evidence that supports the benefit of pharmacy-led medication review in reducing readmission rates among patients discharged from hospital [6,49,50,51].

The impact of the cuts to MedsCheck services on the quality of the Ontario healthcare system is in question, particularly related to the identification and resolution of drug therapy problems. A better understanding of the impact of changes in service delivery on different subgroups of patients is important. At minimum, we identified that patients with diabetes were possibly more impacted by the policy-change with proportionally fewer MCD completed post-policy changes compared to MCH or MC annual. However, it is also possible that pharmacies strategically switched from billing for MCD annual to billing for MC annual among patients with diabetes taking three or more medications to reduce some of the added paperwork specific to MCD. Further exploration of the use of MedsCheck services among patients with diabetes is of interest. Community pharmacists are increasingly involved in patient care through the conduct of regular medication review [52]. In particular, studies from Australia, Canada, the United Kingdom, and the United States show that diabetes education and support delivered through community pharmacies improves care and outcomes [53,54,55]. However, direct clinical evidence of the various types of medication review programs is scarce. There is a need for well-designed, rigorous studies with more sensitive and specific outcomes that consider the effect of community pharmacists’ contributions to reviewing medications on improving health [52,56,57]. Our results provide compelling evidence of the immediate and sustained effects of policy decisions on the level of publicly reimbursed professional community pharmacy-delivered services. In the future, policy makers are encouraged to work closely with healthcare providers and fund research to provide evidence of the best mechanisms for the implementation of new pharmacy services [45,46]. Funding research to consider the benefits and harms of programs is also important instead of abruptly cancelling programs without considering how to better focus program delivery efforts.

Our research was limited in its ability to understand why the policy change had such a dramatic impact on the number of services delivered, or if the changes impacted the quality of service delivery. Future mixed-methods research is encouraged. Focus groups with community pharmacists will provide a deeper understanding of the experience of delivering MedsCheck services pre- and post-2016 policy change, and thus help uncover the reasoning behind reduced levels of service delivery. Survey research of MedsCheck program recipients may help to clarify the impact of changes on the quality of service delivery, as well as patient satisfaction and possible concerns about the decision by the Ontario government to cancel community services outside transitions in care. Finally, semi-structured interviews with policy decision makers and Pharmacy Council may help clarify the rationale behind the 2016 policy changes, as well as more recent changes to pharmacy practice in Ontario.

## 5. Conclusions

The Ontario government instituted a policy change October 2016 that abruptly changed reporting and documentation requirements that was followed by a sharp and sustained decline in community pharmacy MedsCheck services. Our findings highlight the importance of a well-executed implementation strategy for health policy changes to ensure the feasibility and sustainability of professional community pharmacy services. Despite consulting with pharmacy stakeholders through a formal Pharmacy Council to develop a more standardized service, the comprehensive documentation and interprofessional communication processes put in place were associated with a substantial reduction in the number of professional services delivered. The immediate and profound reduction in service delivery speak to the importance and benefits of pilot studies that consider program feasibility in a real-world setting. Better understanding of the impacts of the 2016 policy change on the quality of MedsCheck services and outcomes, as well as potential harms from cancelling many of these services, are needed to inform future policy-decision making.

## Figures and Tables

**Table 1:** MedsCheck documentation requirement pre- and post-October 2016 policy change.

Before October 2016	Since October 2016 Policy Change
*Patient Assessment Summary with Pharmacist’s Signature and Date*	1. *MedsCheck Patient Acknowledgement of Professional Pharmacy Service* * Completed annually for all community MedsCheck services (not required for MCLTC) 2. *Pharmacists Worksheet* * for professional notes, 4 pages 3. *Personal Medication Record*, 1 or more pages (not required for MCLTC) Signed and dated by the pharmacist indicating the date of the consultation Documentation with assurance that the record is an accurate assessment of the patient’s prescription, non-prescription and natural health product usage Table format for: what, why, how and comments for each product Includes evidence that drug therapy problems have been followed-up or have a plan for resolution Records if the optional patient take-home summary was provided 4. *Healthcare Provider Notification of MedsCheck Services* *, 1 page Mandatory summary provided to the primary prescriber that includes the *Personal Medication Record* and lists follow-up issues identified for resolution (not required for MCLTC) 5. *MedsCheck Patient Take-Home Summary* *, 1 or more pages (optional) Summary of discussion, patient goals, suggestions for how to achieve goals, lists of resources, contacts and referrals Specific to MedsCheck Diabetes annual services 6. *Diabetes Education Checklist* *, 4 pages 7. *Diabetes Education Patient Take-Home Summary* *, 1 or more pages Summary of discussion, patient goals, suggestions for how to achieve goals, lists of resources, contacts and referrals Unlike other community MedsCheck services, the take-home summary is required for patients receiving MedsCheck Diabetes services
Specific to MCH *Medicine Cabinet Review*: Review of the medicine storage areas Expired or unused medications itemized removed from the home with signed consent for removal Specific to MCLTC Name of designate of long term care team with whom results reviewed	

* Standardized forms, available at http://www.forms.ssb.gov.on.ca/, accessed on 2 May 2019 [15]; * MCH: MedsCheck at Home, MCLTC: MedsCheck Long-Term Care.

**Table 2:** MedsCheck service administrative codes and reimbursement fees as of October 2016.

Service	PIN	Fee
**MedsCheck (MC) ^1^**		
MC annual	93899979	$60 ^1^
MC follow-up		
• Hospital discharge ≤ 2 weeks	93899981	
• Pharmacist decision	93899982	$25
• Physician or nurse practitioner referral	93899983	
• Planned hospital admission	93899984	
**MedsCheck Diabetes (MCD) ^2^**		
MCD annual	93899988	$75
MCD follow-up	93899989	$25
**MedsCheck at Home (MCH) ^3^**		
MCH annual	93899987	$150
**MedsCheck Long Term Care (MCLTC) ^4^**		
MCLTC annual	93899985	$90
MCLTC quarterly	93899986	$50

^1^ $50 before October 2016, eligible for residents with valid Ontario health card taking 3 or more chronic medications.^2^ eligible for residents with diabetes and a valid Ontario health card. ^3^ eligible for residents with a valid Ontario health card and with diabetes or taking 3 or more chronic medications and unable to attend community pharmacy to have a MedsCheck service. ^4^ eligible for residents of licensed long-term care (LTC) homes with valid Ontario health card and taking 3 or more chronic medications. Pharmacist participation in annual and quarterly medication review services in LTC are required by legislation [13]. PIN: Product Identification Number.

**Table 3:** Demographic information of recipients and total number of Medscheck claims, by service type *.

Service Type	MedsCheck (Annual or Follow-Up)	MedsCheck Diabetes (Annual or Follow-Up)	MedsCheck at Home	MedsCheck Long Term Care (Annual or Quarterly)
Number of recipients	647,740	209,060	57,583	95,191
Number of Service claims	2,952,434	848,911	103,591	1,084,410
Age, mean (SD)	58.3 (17.3)	60.6 (14.2)	74.4 (16.4)	82.6 (10.5)
Women, %	52.4	45.2	62.1	64.0

* Age and sex for each service type based on first service date for that service type. SD = Standard Deviation.

**Table 4:** Results of interrupted time-series segmented regression models, October 2014–September 2018, with intervention (policy change) 1 October 2016.

	MedsCheck		MedsCheck Diabetes		MedsCheck at Home	MedsCheck Long Term Care	
Parameter	Annual	Follow-Up	Annual	Follow-Up		Annual	Quarterly
Total Claims	2,356,615	595,819	664,483	184,428	103,591	285,403	799,007
Monthly Claims							
• Minimum number	22,973	5768	4314	896	1151	4493	14,000
• Maximum number	70,554	21,353	23,443	8304	3482	7074	18,687
**Results of Segmented Regression Models**							
Baseline (October 2014), number (95% CI)	63,223 (60,452, 65,994)	16,698 (15,826, 17,771)	18,984 (18,069, 19,899)	6704 (6157, 7251)	2575 (2454, 2697)	6274 (6199, 6349)	16,848 (16,355, 17,341)
Pre-intervention slope, mean monthly claims (95%CI)	−173 (−373, 27)	67 (−1, 135)	81 (14, 149)	−19 (−57, 19)	15 (6, 24)	−6 (−11, −0.3)	18 (−17, 52)
Level Change at Intervention (October 2016)							
• Number of claims (95% CI)	−28,167 (−32,034, −24,302)	−11,854 (−13,205, −10,503)	−14,876 (−16,244, −13,508)	−5214 (−5942, −4487)	−1635 (−1828, −1442)	−929 (−1034, −825)	−1656 (−2348, −965)
• Relative percent change (95% CI)	−46.7 (−52.9, −40.4)	−64.3 (−71.1, −57.3)	−70.5 (−76.7, −64.3)	−83.2 (−94.3, −72.1)	−55.0 (−61.2, −48.8)	−14.4 (−16.0, −12.8)	−9.4 (−13.2, −5.6)
Post-intervention slope, mean monthly claims (95% CI)	521 (42, 1002)	52 (−110, 215)	139 (−17, 296)	16 (−77, 108)	22 (4, 40)	42 (30, 54)	50 (-33, 132)
Relative difference to forecast (September 2018), proportion (95% CI)	−21.0 (−27.7, −14.3)	−61.0 (−67.3, −54.5)	−58.9 (−64.6, −53.2)	−75.8 (−87.7, −63.8)	−44.9 (−50.4, −39.3)	3.5 (1.8, 5.1)	−5.1 (−8.8, −1.3)

CI: Confidence Interval.
